# The GUL-1 Protein Binds Multiple RNAs Involved in Cell Wall Remodeling and Affects the MAK-1 Pathway in *Neurospora crassa*

**DOI:** 10.3389/ffunb.2021.672696

**Published:** 2021-04-16

**Authors:** Inbal Herold, Avihai Zolti, Marisela Garduño-Rosales, Zheng Wang, Francesc López-Giráldez, Rosa R. Mouriño-Pérez, Jeffrey P. Townsend, Igor Ulitsky, Oded Yarden

**Affiliations:** ^1^Department of Plant Pathology and Microbiology, The Robert H. Smith Faculty of Agriculture, Food and Environment, The Hebrew University of Jerusalem, Rehovot, Israel; ^2^Departamento de Microbiología, CICESE (Centro de Investigación Científica y Educación Superior de Ensenada), Ensenada, Mexico; ^3^Department of Biostatistics, Yale University, New Haven, CT, United States; ^4^Department of Ecology and Evolutionary Biology, Yale University, New Haven, CT, United States; ^5^Yale Center for Genome Analysis, Department of Genetics, Yale University, New Haven, CT, United States; ^6^Department of Biological Regulation, Weizmann Institute of Science, Rehovot, Israel

**Keywords:** GUL-1, RNA-binding protein, cell wall integrity pathway, cell wall remodeling, COT-1, Nikkomycin, MAPK

## Abstract

The *Neurospora crassa* GUL-1 is part of the COT-1 pathway, which plays key roles in regulating polar hyphal growth and cell wall remodeling. We show that GUL-1 is a bona fide RNA-binding protein (RBP) that can associate with 828 “core” mRNA species. When cell wall integrity (CWI) is challenged, expression of over 25% of genomic RNA species are modulated (2,628 mRNAs, including the GUL-1 mRNA). GUL-1 binds mRNAs of genes related to translation, cell wall remodeling, circadian clock, endoplasmic reticulum (ER), as well as CWI and MAPK pathway components. GUL-1 interacts with over 100 different proteins, including stress-granule and P-body proteins, ER components and components of the MAPK, COT-1, and STRIPAK complexes. Several additional RBPs were also shown to physically interact with GUL-1. Under stress conditions, GUL-1 can localize to the ER and affect the CWI pathway—evident via altered phosphorylation levels of MAK-1, interaction with *mak-1* transcript, and involvement in the expression level of the transcription factor *adv-1*. We conclude that GUL-1 functions in multiple cellular processes, including the regulation of cell wall remodeling, via a mechanism associated with the MAK-1 pathway and stress-response.

## Introduction

The *Neurospora crassa* GUL-1 protein (NCU01197) is a component of the COT-1 (Colonial Temperature sensitive 1; NCU07296.7) NDR-kinase pathway (Herold et al., 2019). The components of this pathway play key roles in regulating cell wall remodeling and polar growth, fundamental processes of filamentous fungi (Yarden et al., 1992; Gorovits et al., 2000; Gorovits and Yarden, 2003; Maerz and Seiler, 2010; Ziv et al., 2013; Herold and Yarden, 2017). Impaired function of COT-1 results in significant morphological defects (Yarden et al., 1992; Gorovits et al., 1999; Maerz et al., 2008; Herold and Yarden, 2017). Inactivation of *gul-1* partially suppresses the severity of growth phenotypes associated with *cot-1* (ts) and its coactivator *mob-2a* (Terenzi and Reissig, 1967; Herold and Yarden, 2017; Aharoni-Kats et al., 2018). Deletion of *gul-1* modifies the expression of cell wall remodeling genes (Herold and Yarden, 2017), nitrogen and amino-acid metabolism genes and almost 300 genes of unknown function (Herold et al., 2019). However, no study has established whether GUL-1 physically interacts with these mRNA species and/or directly regulates their translation. The *Aspergillus fumigatus* GUL-1-homolog SsdA also plays an important role in cell wall maintenance. Overexpression of *ssdA* increases chitin levels; loss and overexpression of *ssdA* alter the localization of the *A. fumigatus* chitin-synthase CsmA (Thammahong et al., 2019).

The GUL-1 protein is distributed within the entire cell volume (Lin et al., 2018; Herold et al., 2019) as a soluble protein, and has also been shown to aggregate and undergo microtubule-dependent trafficking. GUL-1 aggregates were found to associate with peri-nuclear areas in a stress-dependent manner (Herold et al., 2019).

The *Saccharomyces cerevisiae* GUL-1 homolog is Ssd1, whose activity is modulated by the yeast COT-1-homolog Cbk1 (Jorgensen et al., 2002; Kurischko et al., 2005; Jansen et al., 2009; Kurischko and Broach, 2017). Ssd1 is an mRNA binding protein (RBP) that physically interacts with 59–152 mRNA transcripts encoding proteins that function in cell wall organization and remodeling (Hogan et al., 2008; Jansen et al., 2009). Ssd1 recognizes conserved motifs in the 5′ and 3′ UTRs that are present in some of the associated mRNAs (Hogan et al., 2008; Ohyama et al., 2010; Wanless et al., 2014). Under stress conditions, Ssd1 has been shown to associate with mRNAs and processing bodies (PBs) at the low-complexity prion-like domain (PLD) and with stress granules (SGs). The outcome of these physical associations is translational repression, storage, or degradation of the bound mRNAs (Tarassov et al., 2008; Kurischko et al., 2011; Richardson et al., 2012; Zhang et al., 2014; Kurischko and Broach, 2017). The core of PBs complexes is comprised of components of the mRNA decapping machinery (Anderson and Kedersha, 2006 and references within) and translational factors. SGs are formed, in response to stress, when translational initiation is impaired. They are comprised of translation initiation factors, poly (A) RBP, and the 40S ribosomal subunit (Decker and Parker, 2012).

Deletion of *ssd1* results in activation of the Cell Wall Integrity (CWI) pathway in a constitutive manner (Arias et al., 2011). Ssd1 functions in parallel to Mpt5 (another RBP) to maintain CWI (Kaeberlein and Guarente, 2002). In *N. crassa*, the conserved CWI pathway is activated under cell-wall stress conditions and modulated by different proteins, including a Mitogen-Activated Protein-Kinase (MAPK) cascade that is comprised of MIK-1 (NCU02234), MEK-1 (NCU06419), and MAK-1 (NCU09842) (Park et al., 2008; Bennett et al., 2013; Kamei et al., 2016). This pathway can alter the transcription of multiple genes via the transcription-factor ADV-1 (NCU07392), encoded by a gene that is conserved among the Pezizomycotina and is essential for germling communication and fusion (Leeder et al., 2013; Dekhang et al., 2017; Fischer et al., 2018). ADV-1 has also been shown to directly activate transcription of elements involved in cell-wall stress response, including *mek-1* (Fischer et al., 2018; Fischer and Glass, 2019).

While we have previously analyzed the changes in the transcriptome changes that occur in conjunction with the presence/absence of GUL-1 (Herold et al., 2019), the emphasis of this report is the demonstration that GUL-1 is a bona fide mRNA-binding protein that associates with multiple mRNA species including *gul-1* itself. We also show that GUL-1 can physically interact with multiple proteins including additional RBPs, translation processes, and CWI pathway components. Lastly, we provide evidence that GUL-1 affects the CWI pathway via altering phosphorylation of MAK-1, and conclude that the RBP functions in the regulation of cell wall remodeling in concert with the MAK-1 pathway in a stress-related manner.

## Materials and Methods

### Strains, Media, and Growth Conditions

*N. crassa* strains used in this study are listed in Table 1. Strains were grown in either liquid or solid (supplemented with 1.5% agar) Vogel's minimal medium with 1.5% (w/v) sucrose (Vs), as described by Davis, 2000) or is available at http://www.fgsc.net/Neurospora/NeurosporaProtocolGuide.htm.

**Table 1:** *Neurospora crassa* strains used.

**Strain**	**Genotype**	**Source**	**NCU number**
Wild type	74-OR23-1 A	FGSC#987	
Wild type	ORS-SL6 a	FGSC#4200	
Δ*gul-1*	Δ*gul-1*	Seiler et al., 2006	NCU01197
GUL-1-GFP	his-3+::Pccg1-gul-gfp A	Seiler lab	
GFP	his-3::Pccg1 gfp	Fleissner et al., 2009	
dsRED-NCA-1	Pccg1::t-dimer2 (12)-nca-1::his-3+;Δmus-51::bar+	Bowman et al., 2009	
GUL-1-GFP/dsRED-NCA-1		This study	

Cultures designated for RNA extraction consisted of 2 × 10^6^ conidia ml^−1^ grown in 20–50 ml of liquid Vs (150 rpm) for 16 h at 34°C. For cell-wall stress induction, cultures were supplemented with 10^−5^ M Nikkomycin Z, for the last hour prior to RNA extraction. RNA extraction, DNase treatment, cDNA, and RT PCR (primers are listed in Table 2) were performed as previously described (Herold and Yarden, 2017).

**Table 2:** Primers used for RT PCR analyses.

**Name**	**NCU number and gene designation**	**Sequence**	**TM**	**Product size (bp)**
gul1_ For1912	NCU01197 (*gul-1*)	GGAAGAGGAGATCAATGATGAGCA	64	81
gul1_ Rev1993		AGCAGACCAAGGGTACCAGAAAAC		
404_For333	NCU05404 (*ghx-2*)	CGCTACCTTCACCGTCATCG	64	122
404_Rev455		CTGTTGTGCCAGGTCCAAGC		
347_For1517	NCU07347 (*gh17-2*)	CGGCACCGATTGTAACCAAA	64	105
347_Rev1622		GTGTTGCCATCCTGCCAGAC		
adv1_ For 1455	NCU07392 (*adv-1*)	CCTTGTTCGCGCACACCTAC	64	133
adv1_ Rev1588		TTCAGCGACTCAAGGCAAGC		

### RNA Immunoprecipitation (RIP) and RIP-RNAseq Analysis

The RIP procedures used here were conceptually based on those described by Dang et al. (2016) and Niranjanakumari et al. (2002). 10^6^ conidia of the GUL-1-GFP strain were cultured in 50 ml of liquid Vs at 150 rpm, for 18 h, at 34°C. This strain was confirmed to maintain its functional activity in *N. crassa* [(Lin et al., 2018). A similar strain was shown to be functional in Sordaria macrospora (Stein et al., 2020)]. The mycelia were harvested under vacuum and ground in liquid nitrogen. Tissue powder of total cell extracts was suspended in 1 mL of ice-cold lysis buffer (25 mM Tris HCl at pH 7.5, 150 mM NaCl, 1.5 mM MgCl2, 1% NP- 40, 1 mM EDTA) supplemented with cOmplete protease inhibitor mixture (Roche Applied Science, Mannheim, Germany), 0.5 mM DTT and 0.1 U/μl Murine RNase inhibitor (MO314S, New England BioLabs, Ipswich, MA). The samples were mixed, by vortex, for 2 min and incubated on ice for 10 min. This mixing was repeated three times. The samples were centrifuged for 20 min, 4,500 rpm (at 4°C) and the supernatants were recovered. The samples were then centrifuged for 60 min at 13,000 rpm. 10 mg of protein (from the recovered supernatants) were suspended in a total volume of 1.2 mL of ice-cold lysis buffer. 25 μL of GFP-Trap magnetic agarose beads (gtma-20, Chromotek) were washed according to the manufacturer's instructions and were added to the samples. The resulting mixtures were subjected to a short, 30 min (at 4°C) incubation on a rotation device, in order to reduce the presence of non-specifically bound proteins prior to the main immunoprecipitation phase of the experiment. After magnet-based removal of the beads from the mixture, 0.1 U/μl Yeast tRNA (AM7119, Invitrogen, Waltham, MA) were added to each sample. 600 μl of these samples were retained for total protein assessment. The remaining 600 μl were transferred to 25 μL of new, pre-washed, GFP-magnetic beads and rotated overnight at 4°C. The beads were then thoroughly washed three times (each time for 10 min) with 500 μL washing buffer (25 Mm Tris HCl at pH 7.5, 300 mM NaCl, 1.5 mM MgCl2, 1% NP-40, 1 mM EDTA) supplemented with cOmplete protease inhibitor mixture, 0.5 mM DTT and 0.1 U/μ RNase inhibitor. The beads were incubated and vortexed with 500 μl TRI reagent (TR118, MRC Cincinnati, OH) for 5 min at room temperature. RNA extraction was performed from the TRI reagent mixture followed by Perfecta DNase I treatment (Quanta BioSciences, Gaithersburg, MD, USA), according to the manufacturer's protocols. cDNA was synthesized with the qScript Flex cDNA kit (Quanta Biosciences, Beverly, MA). RT PCR analyses were performed as previously described (Herold and Yarden, 2017). The abundance of mRNAs was determined by RT PCR along with a no-tag control (wild-type strain) and compared to the abundance determined in total RNA extracts. The analyses were carried out with three independent replicates.

In order to obtain a comprehensive analysis of RNA species bound to GUL-1, we first used the RNA isolation procedure described above for the RIP experiments. A total of 24 qualified samples, including two biological replicates were prepared. The eight treatments analyzed were designated GPU, GPT, GNU, GNT, WPU, WPT, WNU and WNT. These corresponded to GUL-1 (G) or wild type (W), pulldown (P) or non-pulldown (N) with RIP, and either treated (T) or untreated (U) with Nikkomycin Z. Sequencing libraries were produced from purified total RNA samples using the Illumina TruSeq stranded protocol. Messenger RNA was purified using Dynabeads oligo(dT) magnetic separation (Invitrogen). Preparation of cDNA for sequencing followed the Illumina mRNA Sequencing Sample Preparation Guide. Complementary DNA was primed for reverse transcription using N_6_ primers. The quality of cDNA samples was verified with a bioanalyzer (Agilent Technologies). All samples were sequenced at the Yale Center for Genomics Analysis (YCGA).

The libraries underwent 76-bp single-end sequencing using Illumina HiSeq 2500 according to Illumina protocols. One of the three replicates for two sample types (GNU and GNT) produced very low reads due to low concentrations and were excluded from further analyses. The other 22 samples generated an average of 31.8 million single-end reads per library. Adapter sequences, empty reads, and low-quality sequences were removed. For each read, we trimmed the first six nucleotides, and trimmed the last nucleotides at the point where the Phred score of an examined base fell below 20 using in-house scripts. If, after trimming, the read was shorter than 45 bp, the entire read was discarded. Trimmed reads were aligned to the *N. crassa* genome from the NCBI with its genome annotation using Tophat v.2.1.1 (Trapnell et al., 2009), applying the very-sensitive preset and providing the corresponding gene model annotation. Only the reads that mapped to a single unique location within the genome, with a maximum of two mismatches in the anchor region of the spliced alignment, were reported in these results. We used the default settings for all other Tophat options. We tallied reads by aligning to exons of genes with the program HTSeq v0.6.1p1. A tally of the number of the reads that overlapped the exons of a gene, was calculated using aligned reads and the gene structure annotation file for the reference genome. Differentially expressed transcripts were analyzed using the STRING protein association network (Szklarczyk et al., 2015, 2017) with an integrated minimum required interaction score customized to medium confidence (0.4), using Cytoscape integrated app (Shannon et al., 2003). The GLay algorithm was applied for clustering the STRING network landscape using the ClusterMaker2 Cytoscape integrated app (Su et al., 2010; Morris et al., 2011). KEGG pathway enrichment was set to a false-discovery rate *P*-value of 0.05. Other figures were plotted with the R' ggplot2 package (Wickham, 2016). Sequence data were deposited to the Gene Expression Omnibus database (GSE152935; Supplementary Tables 1, 2).

### Immunoprecipitation, Western Blotting, and Mass Spectrometry

10^6^ conidia ml−1 cultures were grown in 50 ml liquid Vs medium (150 rpm) for 18 h at 34°C. When required, the strains were exposed to Nikkomycin Z (as described above) or the Vs medium was replaced medium lacking NH_4_NO_3_ (the main nitrogen source) for the last 1 h of growth. Protein extraction, blotting and mass spectrometry were carried out similarly to that described by Shomin-Levi and Yarden (2017), with appropriate modifications: Mycelia were vacuum-filtered, flash-frozen and ground in liquid nitrogen, and then suspended in approximately 2 ml lysis buffer [0.6 M sorbitol, 10 mM HEPES (pH 7.5), 5 mM EDTA, 5 mM EGTA, 5 mM NaF, 0.1 M KCl, 0.2% Triton X-100, and cOmplete protease inhibitor mixture (Roche Applied Science, Mannheim, Germany)]. When required, 1 tube of phosphatase inhibitor (PhosSTOP, Roche) was added to the lysis buffer (10 ml). The samples were mixed, by vortex, for 2 min and incubated on ice for 10 min. This procedure was repeated three times. The samples were centrifuged for 20 min (4,500 rpm, 4°C) and the supernatants were transferred to new tubes. These were then centrifuged for 60 min (13,000 rpm, 4°C) and the supernatants recovered. 80 μg of protein from each sample were resolved in a 4–15% (w/v) SDS-PAGE gel (mini protean TGX precast gels, Bio-Rad, CA, USA) and transferred to polyvinylidene difluoride (PVDF) membranes (Trans-Blot Turbo 0.2 μm, BioRad, CA). The membranes were immersed in a blocking solution (9% skim milk in TBST buffer) for 4 h at room temperature and washed with TBST buffer. Phosphorylated MAK-1 was visualized using an anti-phospho p44/42 (9101 Cell Signaling Technologies, Danvers, MA) according to the manufacturer's instructions (Rocha et al., 2016). The secondary antibody used was goat anti-rabbit IgG-HRP (SC2030, Santa Cruz Biotechnology, Heidelberg, Germany). As a control, we detected β-tubulin with an anti β-tubulin antibody (Rb PAbto beta tubulin, AB15568, Abcam, Cambridge, MA).

For immunoprecipitation analysis, 1 ml of cleared crude extract was incubated, overnight, in a rotation device, at 4°C, with 25 μl GFP-Trap magnetic agarose beads. The beads were washed three times with 500 μl washing buffer (25 Mm Tris Hcl at pH 7.5, 150 mM NaCl, 1.5 mM MgCl_2_, 1% NP-40, 1 mM EDTA). The immunoprecipitated proteins were eluted by treatment for 10 min at 70°C in sample buffer (3×) and were resolved in a 4–15% (w/v) SDS-PAGE gel (mini protean TGX precast gels, Bio-Rad, CA). Gels were stained with Coomassie Blue. The experiment was carried out with three biological replicates, using the GFP strain (Table 1) as a negative control. The proteins in the gel were reduced with 3 mM DTT in 100 mM ammonium bicarbonate [ABC] (60°C for 30 min), modified with 10 mM Iodoacetamide in 100 mM ABC (in the dark, at room temperature for 30 min) and digested in 10% acetonitrile and 10 mM ABC with modified trypsin (Promega) at a 1:10 enzyme-to-substrate ratio, overnight at 37°C. The resulted peptides were desalted using C18 tips (Homemade stage tips) and were analyzed by LC-MS-MS. The peptides were resolved by reverse-phase chromatography on 0.075 × 180-mm fused silica capillaries (J&W) packed with Reprosil reversed phase material (Dr Maisch GmbH, Germany). They were eluted with linear 60 min gradient of 5–28% 15 min gradient of 28–95% and 15 min at 95% acetonitrile with 0.1% formic acid in water at flow rates of 0.15 μl/min. Mass spectrometry was performed by Q Exactive plus mass spectrometer (Thermo) in a positive mode using repetitively full MS scan followed by collision induced dissociation (HCD) of the 10 most dominant ions selected from the first MS scan.

Mass spectrometry data was analyzed using the MaxQuant software 1.5.2.8 (Mathias Mann's group) vs. the *N. crassa* proteome from the Uniprot database. Oxidation on Methionine and acetylation on the N-terminus were accepted as variable modifications and carbamidomethyl on Cysteine was accepted as static modifications. Minimal peptide length was set to six amino acids and a maximum of 2 miscleavages was allowed. Peptide- and protein-level false discovery rates (FDRs) were filtered to 1% using the target-decoy strategy. Protein table was filtered to eliminate the identifications from the reverse database and from common contaminants, and single peptide identifications. The data was quantified by label free analysis using the same software, based on extracted ion currents (XICs) of peptides enabling quantitation from each LC/MS run for each peptide identified in any of experiments. Statistical analysis of the identification and quantization results was done using Perseus 1.6.7.0 software (Mathias Mann's group). Enriched immunoprecipitated proteins were analyzed and visualized by a heatmap (pheatmap with R; Kolde, 2018) or by STRING network as described for the RIP analysis.

### Fluorescence Microscopy

To observe the localization of GUL-1 with respect to the ER, we constructed a heterokaryon from the GUL-1-GFP and ds-mChFP-NCA-1 (Bowman et al., 2009) *N. crassa* strains (Table 1). A Petri dish with Vs or Vs lacking NH_4_NO_3_ (the main source of nitrogen) was inoculated with conidia from the two strains and incubated for 12 h at 30°C. The margin of the colony was screened for hyphae producing both fluorescent markers. Single cell double labeling analyses were performed as previously described (Herold et al., 2019), using an ECLIPSE Ti-E (Nikon, Japan) inverted microscope equipped with a CSU-x1 (Yokogawa, Japan) Spinning Disk powered by a LU-N4 laser unit (Nikon, Japan) for excitation at 488 and 561 nm for GFP or for dsRED, respectively. An Apo 60x/1.49 N.A. oil immersion objective was used. Images were obtained with an iXon Ultra camera (Andor, UK) and analyzed with NIS-Elements Viewer 4.56 software (Nikon, Japan). Final figures were created with Adobe Photoshop CC 2017 (Adobe Systems Inc., San Jose, CA).

The number of GUL-1-GFP aggregates that were associated with the ER or the cytoplasm was quantified in the subapical distal regions of the hyphae. Measurements were performed along a 50 μm long stretch of the hyphal cell and included the entire hyphal segment diameter. The number and distribution of GUL-1 aggregates was measured in 30 and 24 hyphal segments of cultures grown in the presence and absence of NH_4_NO_3_, respectively.

## Results

### GUL-1 Can Interact With Over 2,000 mRNAs

To identify potential GUL-1-interacting transcripts, we scanned the *N. crassa* transcriptome with a 5′-UTR motif, A[G/U]UCAUUCCUU, identified in some of the *S. cerevisiae* mRNAs that interact with Ssd1 (Hogan et al., 2008; Wanless et al., 2014). We found 15 such transcripts that contain the complete 5′ UTRs motif (11 nt) and 439 that contain the motif with one mismatch (Supplementary Table 3).

To determine whether the identified transcripts and GUL-1 can physically interact, we used *in vivo* RIP analyses. Initially, we analyzed the interactions between GUL-1 and three transcripts. These included *gul-1* itself and two mRNAs that encode proteins involved in cell wall remodeling: glycosyl-hydrolase unclassified family-2 (*ghx-2*) and endo-beta-1,3-glucanase (*gh17-*2). All three transcripts have a close match to the yeast 5′ UTR consensus motif in their 5′ UTRs (Supplementary Table 3). Furthermore, these genes also exhibited high global scores that were obtained with the CatRapid algorithm, used for predicting mRNA-protein interactions (Table 3; Bellucci et al., 2011).

**Table 3:** CatRAPID algorithm results.

**Gene**	**NCU number**	**Description**	**Global Score (0–1)**
*gul-1*	NCU01197	cell wall biogenesis protein Ssd1	1
*ghx-2*	NCU05404	glycosyl hydrolase unclassified family-2	0.6
*gh17-2*	NCU07347	endo-beta-1,3-glucanase	0.47

*Global Score predicts the overall interaction ability of a protein-RNA pair. The value of 1 indicates the highest probability of a predicted interaction*.

**Figure 1:**
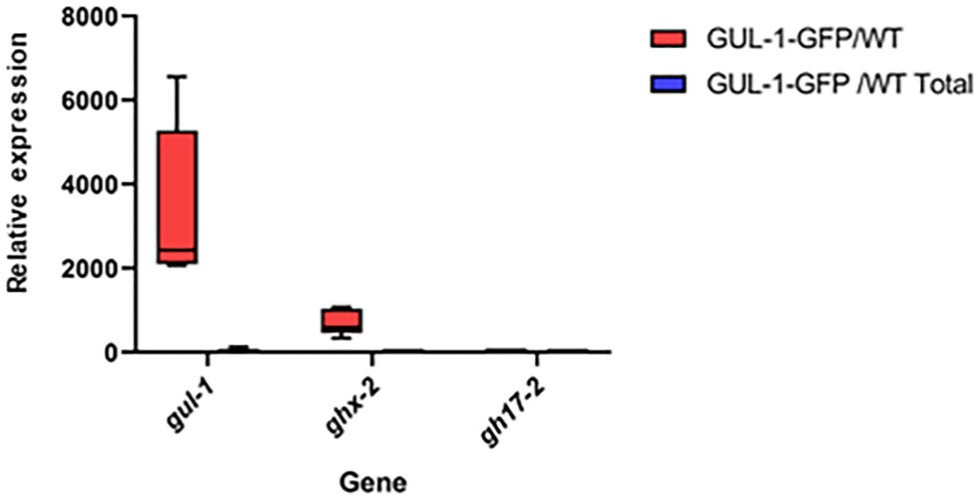
GUL-1 physically interacts with different mRNAs. Relative mRNA abundance of *gul-1, ghx-2*, and *gh17-2*, as determined by RT PCR, in RNA immunopercipitants obtained from the GUL-1-GFP and the wild-type strains. Bars indicate ±1.96 SE (confidence level of 95%), as determined on the basis of three independent replicates.

Based on the ratios obtained between the immunoprecipitated target-gene values and those measured in the total cell extracts (input) obtained from GUL-1-GFP and non-tagged wild-type strains, we inferred interactions between GUL-1-GFP and *gul-1*, as well as *ghx-2* transcripts—yet no interaction with *gh17-2* (Figure 1).

**Figure 2:**
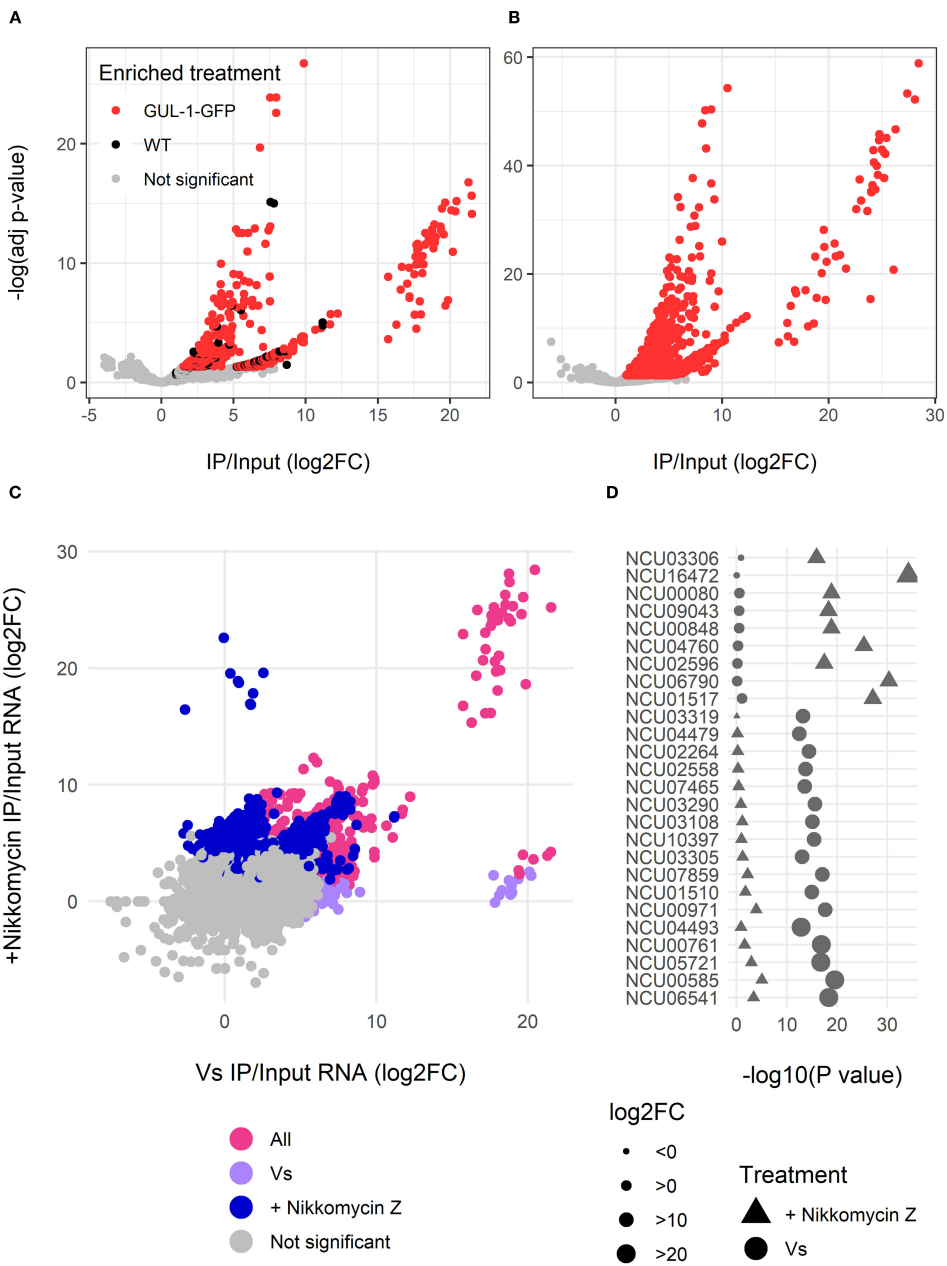
GUL-1 can interact with over 2,000 mRNAs. RNAseq analysis of transcripts immunprecipitated with GUL-1-GFP. Log2 fold-change of GFP pull-down enriched RNAs were compared to wild-type and –Log (adjusted *P*-value) of **(A)** standard growth medium (Vs) and **(B)** Nikkomycin Z treatment. Colors indicate the genes significantly enriched in GFP-labeled GUL-1 (red), but not enriched in wild-type GUL-1 IPs (in black). **(C)** A comparison of enrichment (log2FC) between standard growth conditions (Vs) and Nikkomycin Z treatments was also carried out. Colors indicate transcripts enriched in Vs (magenta), Nikkomycin Z (blue), or in both treatments (pink). Some transcripts were exceptionally enriched in GUL-1 GFP-tagged IPs when compared to wild-type, with a log2 FC > 15, and are grouped within elliptical area. **(D)** The predictive association between highly enriched (log2FC > 15) GUL-1-GFP immunoprecipitated transcripts. The significance of the enrichment, –Log (adjusted *P*-value), for the Vs (circles) or Nikkomycin Z (triangles) treatments, is shown as a comparison for enrichment of the same transcript in the corresponding treatments. Symbol size indicates relative abundance (log FC) of enrichment and the colors signify the enriched treatment as in panel **(C)**. Data for all transcripts can be found in Supplementary Table 4.

Upon verifying that GUL-1 is a bona fide RBP, we expanded our analysis of GUL-1-mRNA interactions by performing RIP analysis combined with RNA-Seq. We analyzed RNA samples obtained from cultures grown under standard growth conditions and samples obtained in the presence of the cell wall-inhibitor Nikkomycin Z. Under standard growth conditions, GUL-1 physically interacts in a significant manner (adjusted *P* < 0.05, log_2_ fold-change (log2FC) > 2) with 1,048 different transcripts which increase in abundance in immunoprecipitated GUL-1-GFP, after reference being made to the input and the non-tagged samples (Figure 2A, Supplementary Table 4). The presence of Nikkomycin Z increased the number of mRNAs associated with GUL-1 by more than 2-fold (2,628 mRNAs), when compared to standard growth conditions. Moreover, these enriched genes had a higher log2FC compared to the samples from standard growth conditions (Figure 2B, Supplementary Table 4).

The core GUL-1-interacting mRNAs were enriched for ribosome, metabolic pathway, oxidative phosphorylation, protein processing in the ER, autophagy, citrate cycle and MAPK KEGG pathways. Enrichment in propanoate metabolism and biosynthesis of antibiotics was unique to standard and stress-challenged cultures, respectively (Figure 2C, Supplementary Figure 1, Supplementary Table 4). Overall, in the presence of Nikkomycin Z, we found more significant enrichment of genes sharing common pathways (Figure 2C, Supplementary Figure 1B).

The GUL-1-bound mRNAs (identified in either or both treatments) were enriched with RNAs involved in protein synthesis (Figure 2C, Supplementary Figure 1, Supplementary Table 4). We also identified a large set of transcripts that encode cell-wall remodeling components. Among them, there were 16 cell wall proteins, 15 glucanosyltransferases (part of them also enriched in the metabolic pathways), α-1,3-glucan synthase [*ags-1* (NCU08132)], all 5 *gel* transcripts (NCU08909, NCU07253, NCU01162, NCU06850 and NCU06781) and a chitin synthase [*chs-1* (NCU03611)]. An association between GUL-1 and at least 17 circadian clock-associated transcripts was evident as well.

Regardless of the growth conditions tested, GUL-1 was found to associate with 828 common transcripts (Figure 2, Supplementary Table 4). These transcripts included the catalytic and regulatory subunits of glucan synthase, *fks-1* (NCU06871) and *rho-1* (NCU01484), respectively, as well as 3 components of the STRIPAK complex [*pp2A-A* (NCU00488), *ham-2* (NCU03727), *ham*-*3* (NCU08741) and *ham*-*4* (NCU00528)]. In addition, GUL-1 was found to interact with the MAPK-pathway related components *mik-1, so* (NCU02794), *mek-2* (NCU04612), and *mak-2* (NCU02393).

The Nikkomycin-Z-challenged growth condition was characterized by 1,800 unique GUL-1-associated transcripts (Figure 2, Supplementary Figure 1, Supplementary Table 4). In this case, GUL-1 was found to interact with several cell-wall remodeling gene transcripts, as well as components of a recently described Spitzenkörper scaffold [*lah-2* (NCU02793) and *cot-1*] and their motor and cargo adaptor-encoding transcripts [*myo-5* (NCU01440) and *spz-1* (NCU02049), respectively). We found an enrichment of several CWI [*mek-1, mak-1*, and *pp-1* (NCU00430)] and osmoregulatory response [*os-4* (NCU03071) and *os-5* (NCU00587)] pathway transcripts and at least 7 ER related transcripts were identified. Circadian clock components, such as *frq* (NCU02265), *fwd-1* (NCU04540), and *ve-1* (NCU01731), were also identified under these growth conditions.

Among the mRNA species that exhibited a complete match or single mismatch to the yeast 5′-UTR motif, only about half were found to bind to GUL-1 (Supplementary Table 3). Nonetheless, several alternative unique and significantly enriched potential UTR motifs were found (Supplementary Figure 2). To what extent these motifs are necessary or sufficient for GUL-1-mRNA binding has yet to be determined. Taken together, our results show that GUL-1 has RNA binding capabilities and can associate with a broad range of mRNAs. Furthermore, they suggest that GUL-1 may have auto-regulatory attributes, based on its ability to bind its own mRNA.

### GUL-1 Protein Interactions

**Figure 3:**
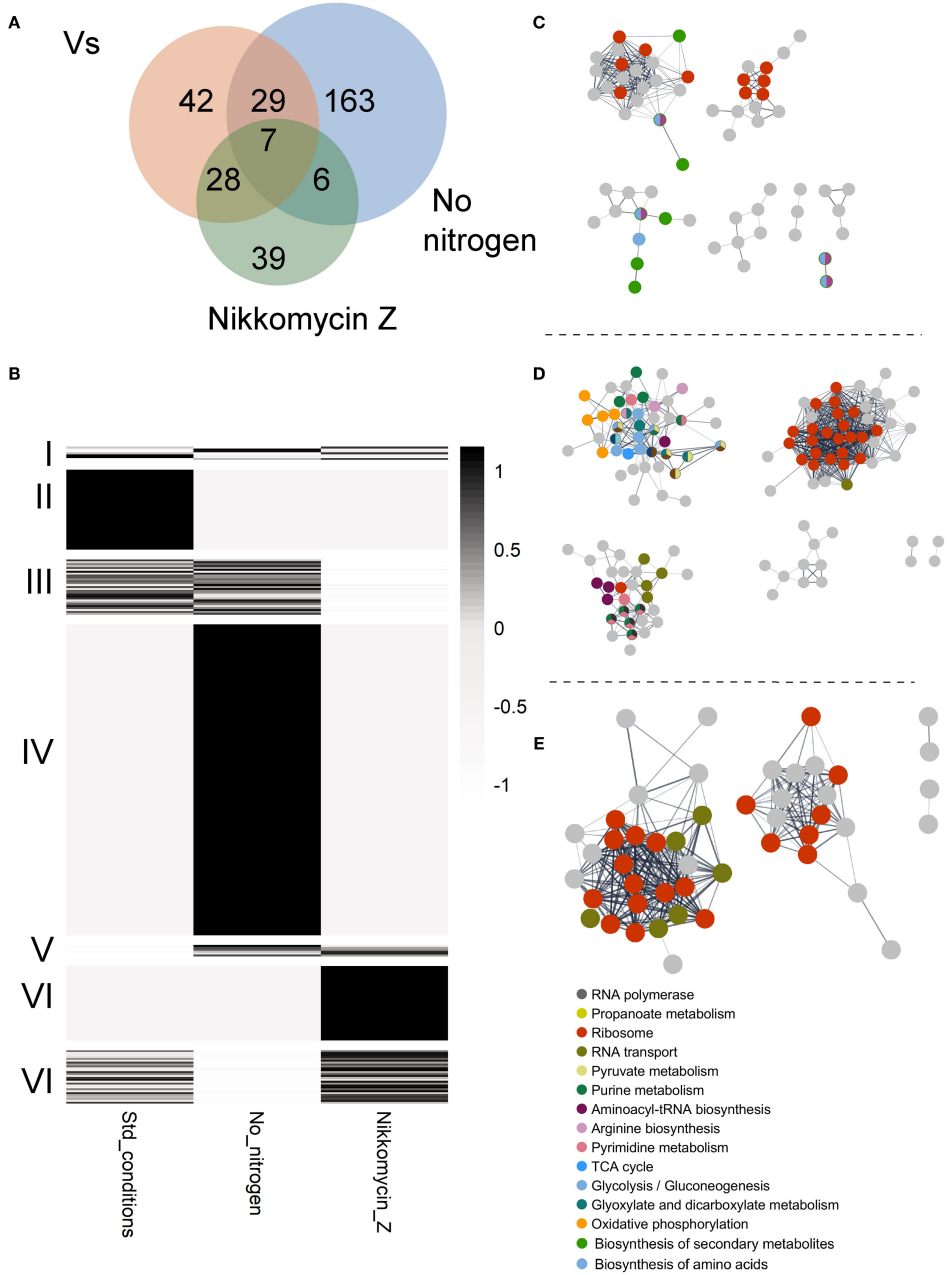
GUL-1 interacts with multiple proteins. **(A)** VENN diagram of specific or shared enriched proteins, associated with the tested treatments. **(B)** A heatmap presenting the average scaled (*z*-score) enrichment, by –log_10_ *P*-value of GUL1 co-immunoprecipitation proteins. The enriched proteins were annotated based on KEGG categories. The proteins were clustered by (I) enrichment for all three treatments, (II) exclusive enrichment in GUL-1 under standard growth conditions, (III) enrichment in both GUL-1 and GUL-1 no nitrogen growth conditions, (IV) enrichment unique to GUL-1 with no nitrogen, (V) shared enrichment between GUL-1 no nitrogen and GUL-1 +Nikkomycin Z growth conditions, (VI) unique enrichment for GUL-1 +Nikkomycin Z, and (VIII) shared enrichment between GUL-1 and GUL-1 + Nikkomycin Z growth conditions. Non-significant observations (*P* > 0.05) were adjusted to 0, to emphasize enriched values. STRING-predicted protein-protein association is presented for enriched proteins of **(C)** GUL-1, **(D)** GUL-1 with no nitrogen, and **(E)** GUL-1 + Nikkomycin Z. Nodes represent enriched proteins, while edges mark predicted associations. Only connected nodes are displayed. The networks were all clustered by GLay community structure algorithms for network clustering. Colored nodes indicate STRING KEGG pathway enrichment. Labeled pathways are at the highest available clustering level (i.e., “Metabolic pathway” or “Carbohydrate metabolism” are redundant when “Glycolysis” pathway is enriched). The full list of enriched categories can be found Supplementary Table 5.

To identify potential GUL-1 interacting proteins, we used protein immunoprecipitation analysis coupled with mass spectrometry. Results were compared to a control strain expressing only the GFP tag (both regulated by the same Pccg1 promotor). Results show that under standard growth conditions, GUL-1 associates with over 100 different proteins (Figure 3A, Supplementary Table 5). Forty-two of these (cluster II) were found to significantly interact with GUL-1 only under standard growth conditions (Figure 3B). Among them, we identified SG proteins such as PAB (NCU04799), PUB (NCU07874), and 40S and the cell-wall remodeling glycosyl-hydrolase GH13-9 (NCU05429). Another RBP, JSN-1 (NCU06199), was also identified under these conditions. The major functional networks enriched under these conditions were ribosomal-related and those involved in primary and secondary metabolism (Figure 3).

We also analyzed changes in the presence of different GUL-1-interacting proteins when the fungus was cultured under stress conditions (nitrogen-limiting conditions, and in the presence of Nikkomycin Z). Interestingly, under all the conditions tested, only a limited, 7-member (cluster I), reoccurring core of GUL-1 associated proteins was observed, and none of whose components appeared to be directly related to stress (Figures 3A,B). Two notable members of the core assembly were another RBP—VTS-1 (NCU00311)—and a C2H2-type zinc-finger domain transcription factor (NCU07952). When nitrogen-limiting conditions were imposed, 205 proteins co-immunoprecipitated with GUL-1. 163 proteins (cluster IV) were unique to these conditions and included several SG proteins (e.g., 11 different 40S subunit proteins). Members of all three MAPK pathways (MAK-1, MAK-2 (NCU02393) and OS-4) as well as the COT-1 and STRIPAK complexes component PP2A [PPH-1 (NCU06630); Yatzkan et al., 1998] were also identified in this treatment analysis. An enrichment of genetic information processing and metabolic pathways included 17 distinct functional groups. Among them, significant enrichment was found in purine metabolism, ribosome, and RNA transport (e.g., eIF3, 4, and 5) networks.

When the fungus was challenged with a CWI pathway-related stress (Nikkomycin Z), 80 proteins were shown to significantly interact with GUL-1. Among them only 39 (cluster VI) were found to specifically interact with GUL-1 in the presence of the antifungal compound (Figure 3, Supplementary Table 5). These 39 included 5 different eIF3, 13 60S, and 2 40S subunit proteins, all of which are considered to be SG components. Another RBP—SCP-160 (NCU03897)—was also found to interact with GUL-1, exclusively in the presence of Nikkomycin Z. Overall, two different pathways were found to be enriched: Ribosomal-related proteins and RNA transport. These two pathways were also enriched under nitrogen limitation.

Taken together, under the stress conditions tested, beyond the core GUL-1 interacting proteins, we identified an additional set of 6 common GUL-1 interacting proteins (cluster V), including a transcription elongation factor (NCU02563), and eIF3G (NCU08046).

**Figure 4:**
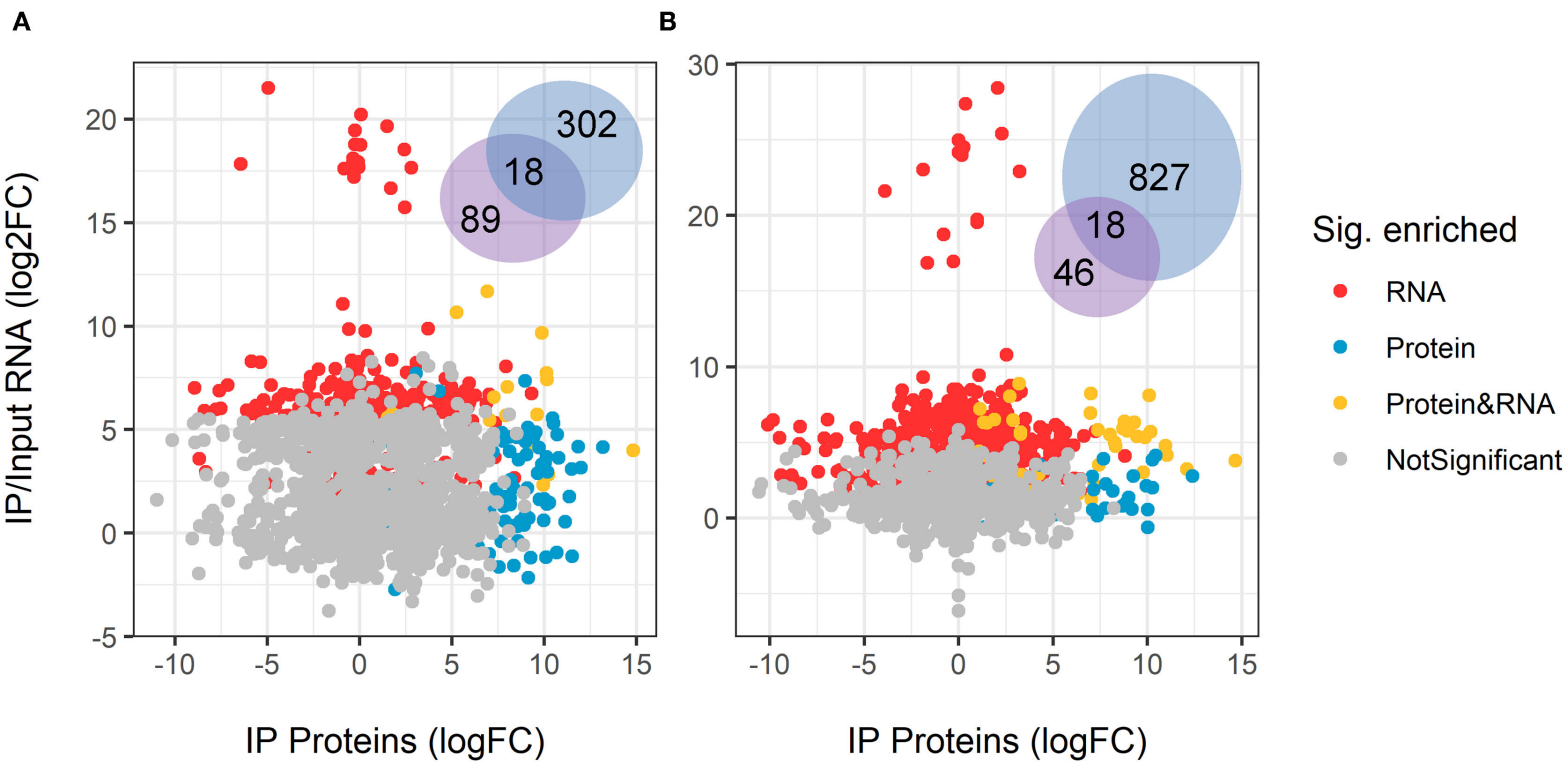
Overlap between enriched GUL-1-immunoprecipitated RNA transcripts and proteins. The log_2_ fold-change of GFP-GUL-1 IP proteins was compared to the enriched RIP RNAseq in **(A)** standard growth conditions (Vs), and **(B)** Nikkomycin Z treatments. No correlation was found for the protein profile and the RNASeq enrichment. Only matching pairs of proteins and RNA transcripts are displayed, when both were detected in the subsequent analysis. The Venn diagram shows the number of shared and uniquely enriched genes or proteins.

When comparing the physical association of GUL-1 with mRNAs and proteins, we observed that under standard growth conditions GUL-1 associated with only 18 common RNA/protein species (Figure 4, Supplementary Table 5). These associations included RBPs such as PUB and VTS-1. In the presence of Nikkomycin Z, we found that 46 of the 81 proteins bound to GUL-1 had corresponding mRNAs that were identified as associated with the RBP. A large number of them were involved in the translation machinery.

### GUL-1 Can Associate With the ER

**Figure 5:**
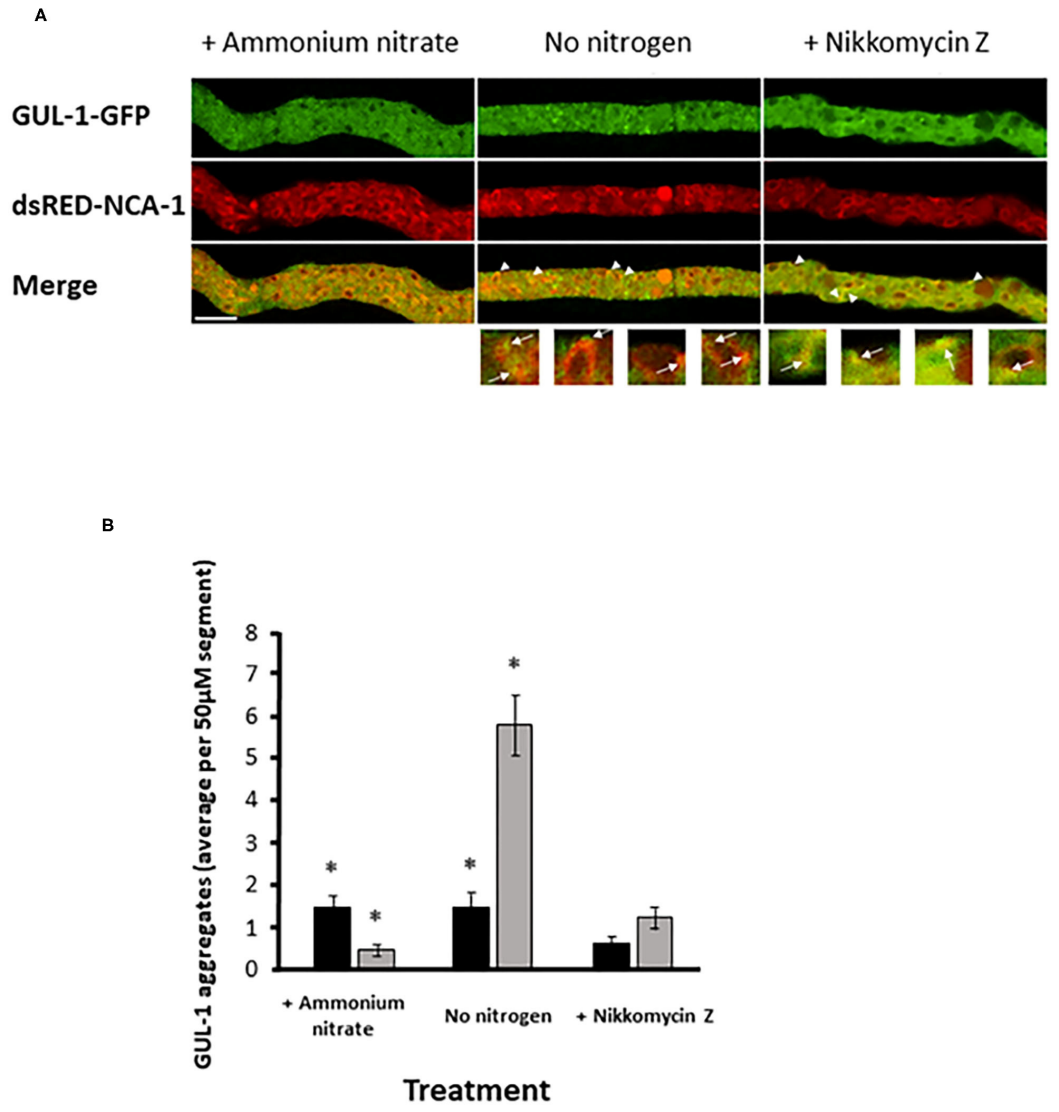
Increase in GUL-1-GFP localization to the ER during stress conditions. GUL-1-GFP aggregates and dsRED-NCA-1 do not appear to co-localize when the fungus is cultured under standard growth conditions (panel **A**, left). When cultures are deprived of nitrogen or exposed to Nikkomycin Z some of the GUL-1-GFP aggregates co-localize with dsRED-NCA-1 (panel **A**; arrowheads and arrows indicate the position of GUL-1-GFP aggregates localized in the ER). Scale bars: 10 μm. **(B)** The number of NCA-1-associated GUL-1-GFP aggregates was determined in hyphal segments of cultures grown in the presence and absence of nitrogen, and also in the presence of Nikkomycin Z (*n* = 30, 24, and 18), Bars indicate standard error. Asterisks indicate significant differences as determined by Student's t-text (*p* ≤ 0.05).

Stress conditions have been shown to increase the incidence of GUL-1 association with the peri-nuclear regions (Herold et al., 2019). As the nuclear envelope was found to be a specialized region of the ER (Newport and Forbes, 1987; Fernández-Ábalos et al., 1998; Prinz et al., 2000; Wedlich-Söldner et al., 2002; Bowman et al., 2009), we probed the possibility that GUL-1 can be localized to this cellular component. To do so, we analyzed the co-localization of GUL-1-GFP and an ER marker - ds-RED-NCA-1 (Bowman et al., 2011). Under standard growth conditions, only about 2 GUL-1-GFP aggregates were observed, on average, in each hyphal segment (Figure 5). Most of these were dispersed in the cytoplasm, while ds-RED-NCA-1 was uniformly distributed around the nuclei. Co-localization of the two tagged proteins in cultures grown under these conditions was rare. In contrast, when nitrogen starvation was imposed, we found ~4-fold more GUL-1 aggregates in each hyphal segment—mostly associated with ER elements (>75%; Figure 5B). A similar trend (_~_65%) was observed when cultures were grown in the presence of Nikkomycin Z (Figure 5B). The increase of GUL-1 interaction with the ER during stress conditions further supports a role in translational regulation during adaptation to environmental challenges.

### GUL-1 Is Involved in the CWI Pathway

**Figure 6:**
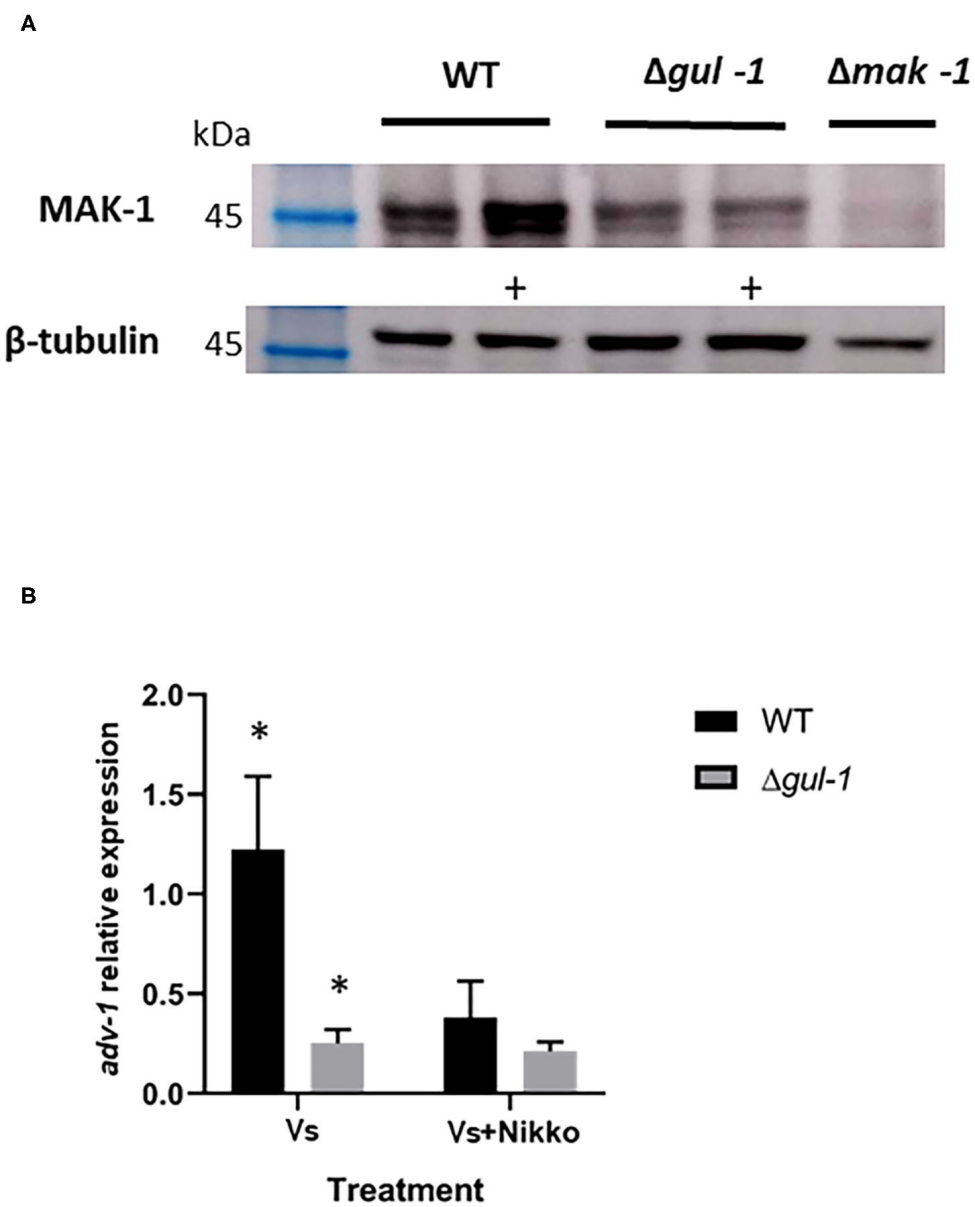
GUL-1 is involved in regulation of the CWI pathway. **(A)** Phosphorylation states of MAK-1 (45 kDa), as determined by probing with anti-phospho p44/42 antibodies, in the wild-type, Δ*gul-1*, and Δ*mak-1* strains under standard growth conditions and 1 h following medium amendment with the chitin synthase inhibitor Nikkomycin Z 10^−5^ M (marked +). β-tubulin (50 kDa) was used as a reference protein. **(B)** Expression profiles of *adv-1* in the wild-type and Δ*gul-1* strains as determined by RT PCR under standard growth conditions and 1 h following medium amendment with Nikkomycin Z (10^−5^ M). The relative expression level of the gene was analyzed by the 2^−ΔCt^ method with the β-tubulin gene as the internal control for normalization (*n* = 3), bars indicate ±1.96 SE (confidence interval of 95%). Asterisks indicate significant differences as determined by Student's *t*-text (*p* ≤ 0.05).

As GUL-1 physically interacts with CWI MAPK pathway components, we examined the consequences on this pathway of eliminating *gul-1*. First, we analyzed the phospho-activation pattern of the MAK-1 protein in the wild-type and in Δ*gul-1* backgrounds under standard growth conditions and in the presence of Nikkomycin Z. In the Δ*gul-1* mutant, the phosphorylation level of MAK-1 was lower than the basal level observed in the wild-type (Figure 6A). Under stress conditions, an expected marked increase in the abundance of phopho-MAK-1 was observed in the wild-type. In contrast, no significant change in phospho-MAK-1 levels were observed after stress was imposed on the Δ*gul-1* culture. We concluded that one of the consequences of inactivation of *gul-1* is compromised function of the CWI pathway in response to stress.

Part of the function of MAK-1 is mediated by the transcription factor ADV-1 (Jonkers et al., 2014; Dekhang et al., 2017; Fischer et al., 2018). We examined the possible effect of deleting *gul-1* on *adv-1* transcription, using RT PCR. Under both standard and Nikkomycin Z stress conditions, expression levels of *adv-1* in the mutant were markedly lower than those measured in the wild-type (Figure 6B). Moreover, under stress conditions, the normal reduction in *adv-1* expression was negligible. These results establish that GUL-1 affects activity of the CWI pathway at different levels and that GUL-1, along with MAK-1, function together in the regulation of cell wall remodeling machinery.

## Discussion

Maintaining polarity is essential for proper hyphal growth and development in filamentous fungi (Riquelme et al., 2011). The process of establishing polarity is highly regulated and involves changes in gene expression, mRNA abundance and localization as well as protein distribution and modifications (Bornens, 2008; Mellman and Nelson, 2008; Holt and Bullock, 2009; Heym and Niessing, 2012; Wang et al., 2019; Kwon et al., 2020). At least part of the post-transcriptional regulatory events involve RBPs, which play critical roles in mRNA processing and fate, such as localization, translation, nuclear export, decay, and gene transcription (Chartrand et al., 2001; Oeffinger et al., 2007; Goler-Baron et al., 2008; Zarnack and Feldbrügge, 2010).

In this study, we have demonstrated that GUL-1 is a bona fide RBP that can bind many different RNA species, including its own mRNA transcript. This autoregulation is in line with that observed for many RBPs (Steinmetz et al., 2001; Roth et al., 2005; Alon, 2007; Hogan et al., 2008), including Ssd1, which binds its own mRNA in a Mg^2+^-dependent manner (Hogan et al., 2008). We showed that GUL-1 can associate with over 2,500 RNAs, implying involvement in diverse processes associated with fungal development (Figure 2). In yeasts, the repertoire of mRNAs bound to Ssd1 represented ~2.5% of the genome. The number of mRNAs bound to the *N. crassa* RBP under standard growth conditions was about 10-fold higher, corresponding to about 10% of the *N. crassa* transcriptome. This fraction was even higher under stress conditions. These results can be at least partly understood as a consequence of the higher complexity and regulation of growth and development of a filamentous fungus.

While identification of the mRNA species that can be bound to GUL-1 was carried out in this study, the mechanistic nature of the physical interactions between GUL-1 and different mRNAs has yet to be resolved. It is likely that at least some of the interactions mentioned throughout this study may be mediated via additional proteins (RBPs and others) that, themselves, interact with GUL-1. Furthermore, it is possible that the GFP tag associated with GUL-1 may have affected some of the kinetics of RNA binding via a change in GUL-1 folding, the GFP tag or the combined chimeric structure.

The number of *N. crassa* genes that contain the RBP-binding motifs which are similar to the *S. cerevisiae* and *S. pombe* consensus sequences (Hogan et al., 2008; Wanless et al., 2014; Nuñez et al., 2016) is limited. This limited overlap of motifs implies that additional, yet unidentified, motifs are key for the mRNA-protein interactions in the filamentous fungus (Supplementary Figure 2). Future analyses will be employed to determine the actual sequence/structural basis for the binding observed here.

As GUL-1 has been previously shown to be involved in cell wall remodeling, it was not surprising to find that many RNAs involved in cell wall biosynthesis and maintenance were found to be associated with this RBP. Ssd1 was also shown to associate with cell wall-related mRNAs, some of which encode proteins that are similar to those bound by GUL-1 [e.g., SUN4 (45% identity to NCU02668), SIM1 (42% identity to NCU002668), and UTH (42% identity to NCU02668) (Hogan et al., 2008; Jansen et al., 2009)].

GUL-1 is a component of the COT-1 pathway. One of the developmental regulatory modules suggested to interact with the COT-1 pathway is the STRIPAK complex (Kück et al., 2019), as has been described for STRIPAK-NDR kinase interactions in additional systems (Duhart and Raftery, 2020). While in *Podospora anseria* GUL-1 is not an integral subunit of STRIPAK, it has been shown to be one of the numerous STRIPAK phosphorylation targets (Stein et al., 2020). Here, we have found that GUL-1 can bind mRNAs that encode components of the STRIPAK complex, providing a novel facet of COT-1-STRIPAK interactions. A functional link between the complexes is further supported by the association of both GUL-1 and STRIPAK with components of the MAK-1 pathway and the localization of STRIPAK and GUL-1 to the nuclear envelope (Dettmann et al., 2013; Herold et al., 2019).

In this study, we found over 1,500 mRNAs whose association with GUL-1 was unique to cultures grown in the presence of Nikkomycin Z. Under these conditions, GUL-1 was found to bind the *cot-1* mRNA and additional Spitzenkörper scaffold-related mRNAs (Ramírez-del Villar et al., 2019; Zheng et al., 2020). This binding is consistent with previous observations that when GUL-1 is more abundant and apparently active, it functions as a negative regulator of COT-1 (which can be found in the Spitzenkörper), which inactivates the RBP by phosphorylation, as demonstrated in the yeast system (Jorgensen et al., 2002; Kurischko et al., 2005; Jansen et al., 2009; Kurischko and Broach, 2017). The changes in mRNA binding under different conditions can be attributed to (i) environmental cues that alter the native transcriptional response and concomitant changes in mRNA abundance, (ii) stress-related modification of GUL-1 binding efficacy (Grammatikakis et al., 2017), and (iii) alterations in the overall RBPome whose members are either recruited or become less active (Garcia-Moreno et al., 2019).

The increased aggregation of GUL-1 under stress conditions and its movement along cytoskeletal elements is also reflected in the profile of mRNAs associated with the RBP. These associations include mRNAs that are related to endosomes and trafficking (Kwon et al., 2020), consistent with the endosome-associated function of some RBPs—including the *P. anserina* GUL-1 (Baumann et al., 2012; Kwon et al., 2020; Stein et al., 2020).

GUL-1 can also physically interact with an array of proteins, among them a large number of members of the translation machinery and RBPs. As expected, GUL-1 was found to associate with two RBPs that are part of the SGs complex: PAB-1 and PUB-1, even though in yeast this interaction was detected only under stress conditions (Kurischko et al., 2011; Hogan et al., 2015; Kurischko and Broach, 2017). GUL-1 also associates with proteins involved in development, such as SNF-5 (NCU02076), a component of the RAM network in *C. albicans* (Finkel et al., 2012) and in *N. crassa* cell fusion (Fu et al., 2014) and VSD-9 (NCU07952) whose absence confers defects in sexual development (Carrillo et al., 2017). Some of the proteins detected represent the networks which we have previously identified to be altered at the transcriptional level as influenced by the presence or absence of *gul-1* (Herold et al., 2019). The association of proteins with GUL-1 is dependent, at least in part, on the culture conditions. For example, the core of GUL-1 interacting proteins appears to be expanded from 7 (under standard growth conditions) to a common 13 when stress is imposed.

One of the apparent core GUL-1 complex components is the posttranscriptional RBP VTS-1, whose mRNA is also bound by GUL-1. The yeast Vts1 has been shown to be involved in mRNA decay and repression (Aviv et al., 2003; Rendl et al., 2008, 2012; She et al., 2017). In *N. crassa vts-1* appears to be essential (Colot et al., 2006).

Under Nikkomycin Z stress conditions GUL-1 had exclusive associations with the SCP-160 RBP and its mRNA. This RBP functions as a negative regulator of PBs formation at the ER and, by that, enhances elongation efficiency (Hirschmann et al., 2014; Weidner et al., 2014). This enhancement is in line with our observation of the physical interaction between GUL-1 and the ER, along with the diverse set of mRNAs encoding ER-related proteins that bind to GUL-1.

The physical interactions between GUL-1 and other RBPs, as found in this study, are consistent with associations that have been previously described in fungi as well as higher eukaryotes (e.g., Sesma, 2016; Zhang et al., 2018). The possibility for both unique and overlapping functions of different RBPs that reside in a single complex has been demonstrated in the case of the *U. maydis* Grp1 and Rrm4 RBPs (Olgeiser et al., 2019). Such a redundancy provides an explanation for why deletion of *gul-1*—in spite of its apparent core role in binding multiple mRNAs—does not result in a severe growth or developmental phenotype. Identification of GUL-1-functionally overlapping RBPs will provide the possibility for further analysis of the RNA and protein – GUL-1 interactome. Such analyses can further validate the mechanistic significance of RNAs and proteins that we have found to interact with GUL-1. Such further analysis would also include assays to distinguish between direct GUL-1 binding proteins and those which interact with GUL-1 via an intermediate. These can potentially include other proteins which are part of the GUL-1 complex, those which are involved in GUL-1 modification or RNA species that are bound to GUL-1 and can act as a scaffold to which additional proteins are attached.

Seven eIF3 subunits (corresponding to NCU00040, NCU05889, NCU01021, NCU08046, NCU07929, NCU09707, and NCU02813) were found to co-immunoprecipitate with GUL-1 as along with some of their mRNAs. The eIF3 plays critical roles in translation (Smith et al., 2013; Merrick and Pavitt, 2018). The association of GUL-1 with eIFs is consistent with its known interaction with a number of SG and PB proteins that have been linked with stress (Ivanov et al., 2019). This association supports our previous suggestions concerning the nature of the GUL-1 aggregates (Herold et al., 2019). Based on the function of PBs and SGs, as determined in other systems and in our current findings, we propose that GUL-1 plays a role in translational repression.

GUL-1 was found to interact with the representatives (mRNAs and the proteins) of all 3 MAPK pathways. This interaction implies that GUL-1 may link functions of distinct pathways in *N. crassa*. We have shown that GUL-1 affects the phospho-activation pattern of MAK-1, and have thus concluded that one of the consequences of inactivation of *gul-1* is compromised function of the CWI pathway in response to stress. One possibility is that the reduced levels of phospho-MAK-1 are a consequence of reduced abundance of the kinase in a Δ*gul-1* background. Another possibility is that MAK-1 turnover is more rapid in a Δ*gul-1* strain. As phosphorylation of MAK-1 requires the cell-wall protein sensors WSC-1 (NCU06910), HAM-7 (NCU00881) (Maddi et al., 2012)—and proper function of various genes such as *mik-1, mek-1, nrc-1, ham-6, mob-3, ham-3* (Maddi et al., 2012; Leeder et al., 2013; Fu et al., 2014; Teichert et al., 2014)—it is possible that GUL-1 is involved in their regulation as well. As the majority of transcripts of these genes are bound by GUL-1, some of this regulation is likely to be direct. Alternatively, there may be a reduction in the abundance of the upstream kinase, MEK-1, as a result of GUL-1 inactivation or an increase in the activity of the opposing phosphatase. Interestingly, in contrast to our observations on the reduced levels of phosphorylated MAK-1 in *N. crassa* in the Δ*gul-1* strain, absence of *ssd1* results in activation of the CWI pathway in a constitutive manner in *S. cerevisiae* (Arias et al., 2011) indicative of at least some functional deviation between the yeast and filamentous fungal homologs.

Our results showing the presence of a physical interaction between the GUL-1 protein and the *mik-1, mek-1*, and *mak-1* transcripts suggest that GUL-1 affects the CWI pathway of *N. crassa* via translational regulation. Involvement of an RBP in the CWI pathway has also been demonstrated in *S. pombe*. There, the RBP Rnc1 was found to bind and stabilize the transcript of the Pmp1 phosphatase, which can dephosphorylate Pmk1 (the MAK-1 homolog). In turn, Pmk1 can activate the Rnc1 RBP by phosphorylation (Sugiura et al., 2003; González-Rubio et al., 2019). In *N. crassa*, part of the function of MAK-1 in regulating gene expression is mediated by the transcription factor ADV-1 (Jonkers et al., 2014; Dekhang et al., 2017; Fischer et al., 2018), whose transcript levels are significantly diminished in the Δ*gul-1* mutant. As no physical interaction between GUL-1 and *adv-1* or ADV-1 were detected, we suggest that the observed effects of GUL-1 on *adv-1* expression are likely mediated via the MAPK pathway. Overall, we conclude that GUL-1 affects activity of the CWI pathway in the fungus at transcriptional, post-transcriptional and post-translational levels and that GUL-1, along with MAK-1, functions to regulate at least some components of the cell-wall remodeling machinery. Whether the phosphoprotein GUL-1 is also regulated by MAK-1 phosphorylation—as part of a regulatory loop—remains to be elucidated.

## Data Availability Statement

The datasets presented in this study can be found in online repositories. The names of the repository/repositories and accession number(s) can be found in the article/Supplementary Material.

## Author Contributions

IH: investigation, methodology, and writing original draft. AZ, MG-R, and ZW: investigation and methodology. FL-G: analysis and methodology. RM-P: supervision and methodology. JT and IU: analysis and methodology. OY: conceptualization and supervision. All authors took part in reviewing and editing of the manuscript.

## Conflict of Interest

The authors declare that the research was conducted in the absence of any commercial or financial relationships that could be construed as a potential conflict of interest.

## Supplementary Material

Supplementary Figure 1 Predictive association between RIP enriched genes. STRING interaction networks are presented for **(A)** standard growth conditions and **(B)** Nikkomycin Z treatments. Each node represents enriched RNA transcripts, while edges mark predictive association. Only nodes connected by >1 edge are displayed. The network was clustered by GLay algorithm (see Materials and Methods) or layout by Y-organic layout (for **A**) and Compound spring embbeder (CoSE) layout (in **B**). Colors indicate KEGG pathways significantly enriched in the network landscape. When a redundancy occurred in the KEGG enrichment analysis, the higher hierarchy category was labeled. The full functional enrichment, including all categories and gene list, can be found in Supplementary Table 4.

Supplementary Figure 2 Enrichment and significance of 5' and 3' UTR motifs as potential binding sites in Gul-1-associated mRNAs.

Supplementary Table 1 Sequence data summary.

Supplementary Table 2 RNASeq gene count.

Supplementary Table 3 *N. crassa* transcripts containing the complete or one mismatch of the yeast Ssd1-binding 5' UTR motif.

Supplementary Table 4 *N. crassa* RNA species that interact with GUL-1.

Supplementary Table 5 *N. crassa* proteins that interact with GUL-1 and common proteins and RNAs that interact with the RBP.
