# Supplementary material for: The GUL-1 Protein Binds Multiple RNAs Involved in Cell Wall Remodeling and Affects the MAK-1 Pathway in Neurospora crassa
Source: Front Fungal Biol. 2021 Apr 16;2:672696. doi: 10.3389/ffunb.2021.672696 (PMC10512220; doi:10.3389/ffunb.2021.672696)
Supplement: Supplementary Figure 1 — Predictive association between RIP enriched genes. STRING interaction networks are presented for (A) standard growth conditions and (B) Nikkomycin Z treatments. Each node represents enriched RNA transcripts, while edges mark predictive association. Only nodes connected by >1 edge are displayed. The network was clustered by GLay algorithm (see Materials and Methods) or layout by Y-organic layout (for A) and Compound spring embbeder (CoSE) layout (in B). Colors indicate KEGG pathways significantly enriched in the network landscape. When a redundancy occurred in the KEGG enrichment analysis, the higher hierarchy category was labeled. The full functional enrichment, including all categories and gene list, can be found in Supplementary Table 4. [file Image_1.pdf]

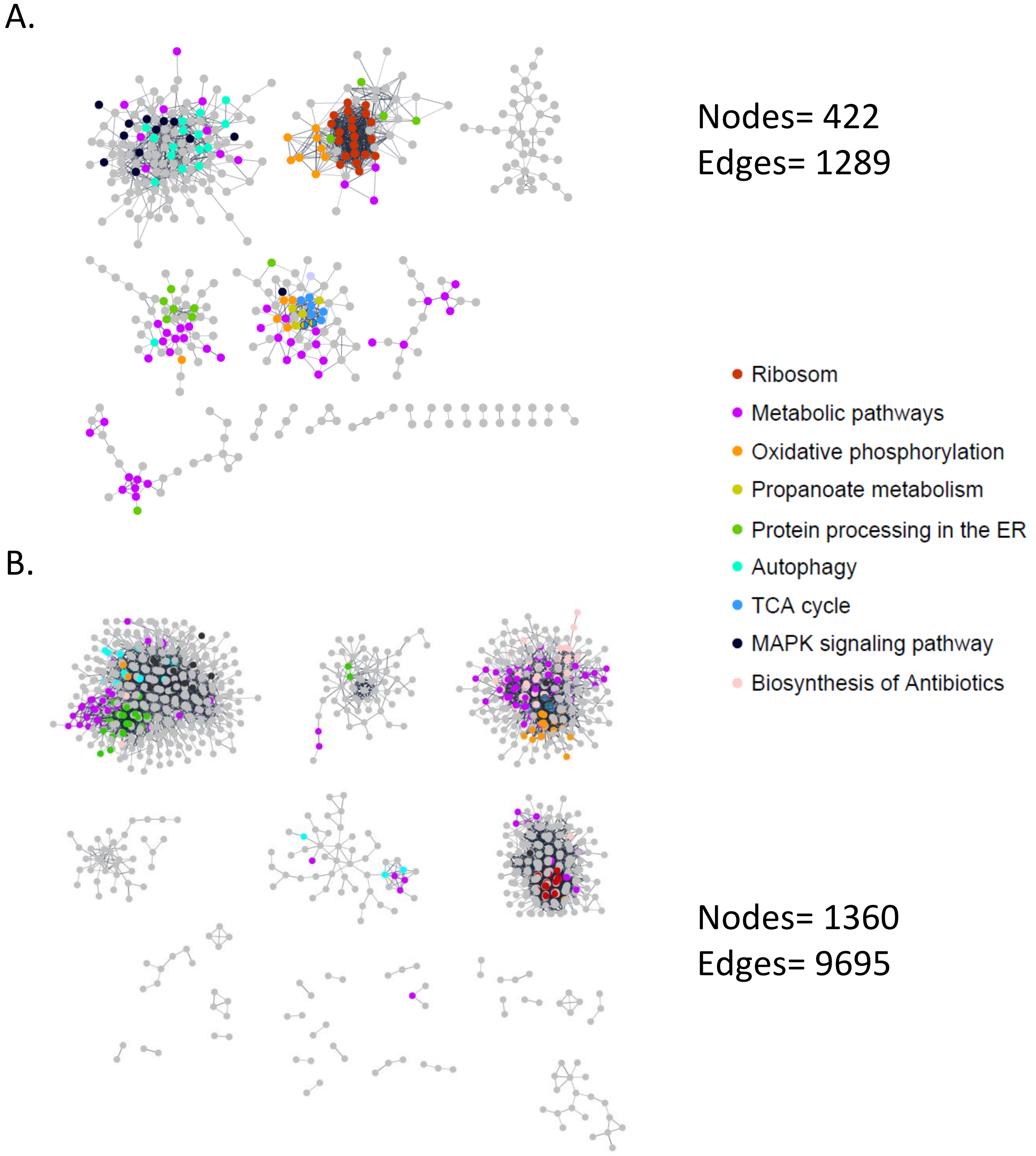

Herold *et al.*, Figure S1
