# Supplementary material for: The GUL-1 Protein Binds Multiple RNAs Involved in Cell Wall Remodeling and Affects the MAK-1 Pathway in Neurospora crassa
Source: Front Fungal Biol. 2021 Apr 16;2:672696. doi: 10.3389/ffunb.2021.672696 (PMC10512220; doi:10.3389/ffunb.2021.672696)
Supplement: Supplementary Figure 2 — Enrichment and significance of 5' and 3' UTR motifs as potential binding sites in Gul-1-associated mRNAs. [file Image_2.pdf]

DREME

Discriminative Regular Expression Motif Elicitation

For further information on how to interpret these results or to get a copy of the MEME software please access http://meme.nbcr.net.

If you use DREME in your research please cite the following paper:

Timothy L. Bailey, "DREME: Motif discovery in transcription factor ChIP-seq data", *Bioinformatics*, **27**(12):1653-1659, 2011. [full text]

DISCOVERED MOTIFS | INPUTS & SETTINGS | PROGRAM INFORMATION

DISCOVERED MOTIFS

Next Top

	Motif	Logo	E-value	Unerased E-value	More	Submit/Download
1.	TCCSC	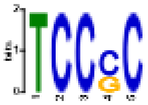	8.3e-076	8.3e-076	↧	--->
2.	CCCTD	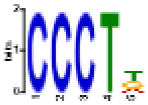	1.1e-069	2.4e-059	↧	--->
3.	CCCVC	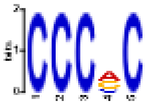	3.0e-071	8.7e-065	↧	--->
4.	CTCST	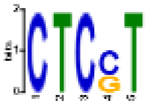	1.4e-069	1.8e-070	↧	--->
5.	VCGCC	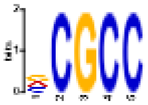	2.3e-067	1.5e-064	↧	--->
6.	CCHGT	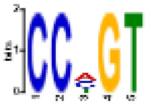	3.4e-067	5.6e-066	↧	--->
7.	TYTCC	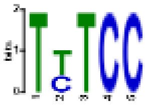	8.7e-065	1.4e-069	↧	--->
8.	BCTCT	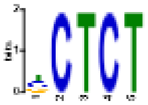	4.5e-063	1.2e-056	↧	--->
9.	CGRSC	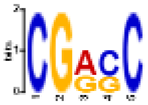	4.3e-061	7.3e-057	↧	--->
10.	CCTDG	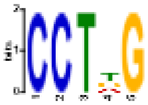	8.7e-059	7.4e-066	↧	--->
11.	CTGBC	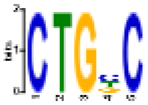	6.4e-056	1.4e-062	↧	--->

	Motif	Logo	E-value	Unerased E-value	More	Submit/Download
**12.**	CTCY	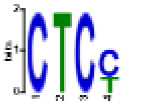	8.1e-054	1.6e-034	↧	--->
**13.**	CGWCK	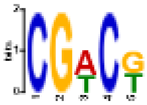	1.6e-054	2.0e-050	↧	--->
**14.**	GCTDC	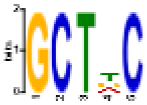	2.5e-053	6.7e-056	↧	--->
**15.**	TACCD	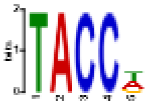	3.3e-049	7.9e-044	↧	--->
**16.**	CCCB	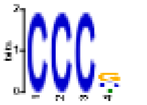	4.1e-049	1.2e-039	↧	--->
**17.**	GCASC	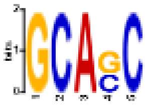	1.2e-049	3.1e-055	↧	--->
**18.**	AGSG	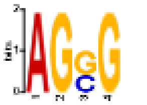	1.0e-048	9.1e-048	↧	--->
**19.**	GGBT	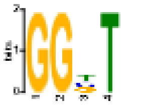	1.0e-050	5.1e-054	↧	--->
**20.**	CDCGC	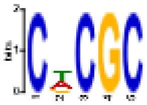	5.3e-045	2.0e-057	↧	--->
**21.**	CTSG	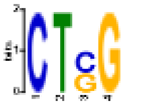	1.1e-046	7.7e-034	↧	--->
**22.**	TCMKT	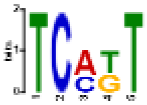	2.1e-043	2.8e-041	↧	--->
**23.**	YGCY	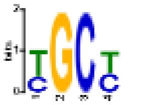	6.7e-048	5.3e-028	↧	--->
**24.**	TYGG	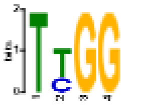	1.6e-041	6.1e-038	↧	--->
**25.**	CCKA	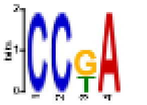	1.9e-040	2.5e-030	↧	--->
**26.**	AGG	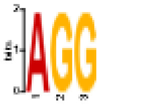	4.4e-040	2.0e-033	↧	--->
**27.**	GGK	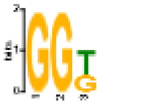	2.6e-040	1.5e-025	↧	--->

	Motif	Logo	E-value	Unerased E-value	More	Submit/Download
**28.**	TCCM	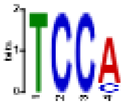	2.2e-039	2.2e-020	↧	⇢
**29.**	GCC	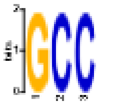	2.3e-037	1.0e-017	↧	⇢
**30.**	CCWC	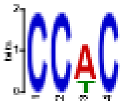	7.5e-039	3.9e-019	↧	⇢
**31.**	CAGY	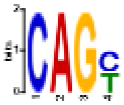	5.1e-037	1.8e-034	↧	⇢
**32.**	CSCA	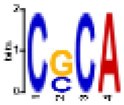	7.4e-038	3.3e-028	↧	⇢
**33.**	YCC	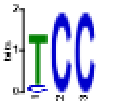	9.3e-040	7.2e-005	↧	⇢
**34.**	CCT	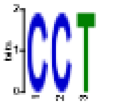	6.0e-039	2.4e-015	↧	⇢
**35.**	GTBG	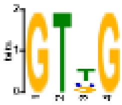	8.0e-038	9.2e-036	↧	⇢
**36.**	GCAD	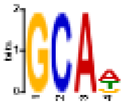	3.5e-038	1.8e-023	↧	⇢
**37.**	KTGY	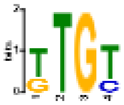	2.4e-037	9.7e-029	↧	⇢
**38.**	DACG	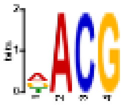	3.1e-036	2.0e-026	↧	⇢
**39.**	DGC	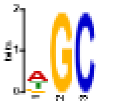	1.1e-035	1.9e-006	↧	⇢
**40.**	GSA	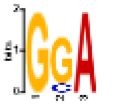	5.4e-035	1.0e-008	↧	⇢
**41.**	CWTT	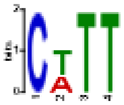	8.6e-035	5.9e-018	↧	⇢
**42.**	TCKA	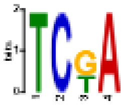	8.8e-035	2.0e-025	↧	⇢
**43.**	TCG	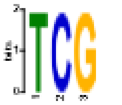	5.1e-038	5.5e-021	↧	⇢

	Motif	Logo	E-value	Unerased E-value	More	Submit/Download
**44.**	TTTK	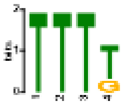	1.3e-033	6.7e-029	↧	--->
**45.**	AAMAA	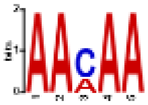	4.2e-032	6.2e-028	↧	--->
**46.**	CVG	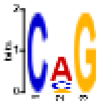	5.6e-032	1.0e-005	↧	--->
**47.**	TTT	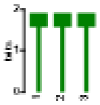	2.7e-031	4.6e-013	↧	--->
**48.**	MGAS	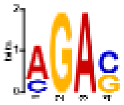	2.7e-031	1.9e-016	↧	--->
**49.**	GTS	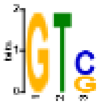	7.7e-031	3.6e-013	↧	--->
**50.**	MGT	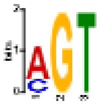	9.4e-032	5.0e-012	↧	--->
**51.**	TTC	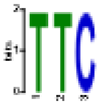	2.4e-031	8.3e-009	↧	--->
**52.**	WYACA	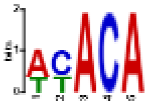	2.5e-030	2.1e-018	↧	--->
**53.**	BACM	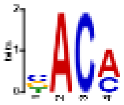	1.8e-030	4.4e-002	↧	--->
**54.**	ACS	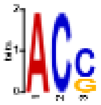	9.4e-033	2.7e-005	↧	--->
**55.**	CSA	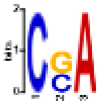	7.8e-030	3.5e-002	↧	--->
**56.**	BACT	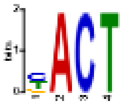	6.0e-029	4.4e-014	↧	--->
**57.**	STT	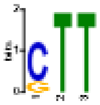	2.3e-031	1.5e-005	↧	--->
**58.**	YTA	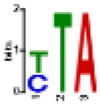	2.1e-031	1.7e-010	↧	--->
**59.**	TST	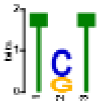	1.0e-028	2.1e-005	↧	--->

	Motif	Logo	E-value	Unerased E-value	More	Submit/Download
**60.**	CDC	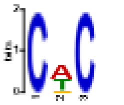	4.1e-029	1.0e-001	↧	⇢
**61.**	RCT	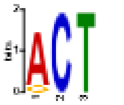	8.5e-026	2.5e-007	↧	⇢
**62.**	VAAR	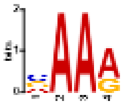	2.1e-025	2.1e-013	↧	⇢
**63.**	DAC	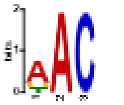	1.5e-027	8.4e-002	↧	⇢
**64.**	ACA	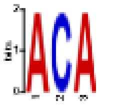	4.1e-027	1.3e-008	↧	⇢
**65.**	RAA	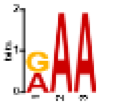	5.4e-026	7.9e-012	↧	⇢
**66.**	CAT	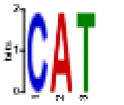	2.7e-024	9.3e-009	↧	⇢
**67.**	TCA	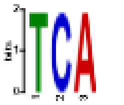	1.8e-024	9.1e-008	↧	⇢
**68.**	YAA	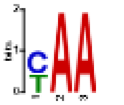	7.1e-025	7.5e-006	↧	⇢
**69.**	AWT	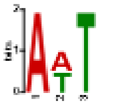	1.8e-025	6.7e-012	↧	⇢
**70.**	WGA	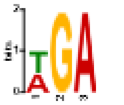	9.5e-025	1.7e-008	↧	⇢
**71.**	KAK	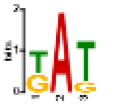	3.9e-026	2.7e-007	↧	⇢
**72.**	RTA	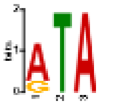	1.6e-024	5.8e-014	↧	⇢
**73.**	YTG	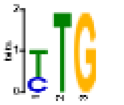	4.1e-023	1.5e-012	↧	⇢
**74.**	ATS	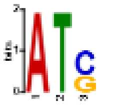	3.7e-019	2.0e-008	↧	⇢
**75.**	GCG	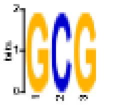	3.8e-011	2.1e-028	↧	⇢

	Motif	Logo	E-value	Unerased E-value	More	Submit/Download
**76.**	AAG	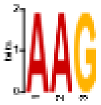	1.9e-009	5.7e-019	↧	⇢
**77.**	TGG	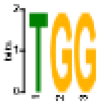	2.4e-007	8.2e-032	↧	⇢

Inputs & Settings

Previous Next Top

Sequences

Source	Alphabet	Sequence Count
untreated.5utr.fa	DNA	1184

Control Sequences

Source	Sequence Count
notenriched.5utr.fa	5293

Background

Name	Bg.				Bg.	Name
Adenine	0.246	A	~	T	0.247	Thymine
Cytosine	0.305	C	~	G	0.202	Guanine

Other Settings

**Strand Handling**	Only the given strand is processed
**# REs to Generalize**	100
**Shuffle Seed**	1
**E-value Threshold**	0.05
**Max Motif Count**	No maximum motif count.
**Max Run Time**	No maximum running time.

Previous Top

DREME version
4.12.0 (Release date: Tue Jun 27 16:22:50 2017 -0700)

Reference
Timothy L. Bailey, "DREME: Motif discovery in transcription factor ChIP-seq data", *Bioinformatics*, **27**(12):1653-1659, 2011. [full text]

Command line

```
dreme -dna -p untreated.5utr.fa -n notenriched.5utr.fa -norc -oc untreated_5utr
```
